# Microstructural, Densitometric and Metabolic Variations in Bones from Rats with Normal or Altered Skeletal States

**DOI:** 10.1371/journal.pone.0082709

**Published:** 2013-12-17

**Authors:** Andrew N. Luu, Lorenzo Anez-Bustillos, Shima Aran, Francisco J. Araiza Arroyo, Vahid Entezari, Claudio Rosso, Brian D. Snyder, Ara Nazarian

**Affiliations:** 1 Center for Advanced Orthopaedic Studies, Beth Israel Deaconess Medical Center and Harvard Medical School, Boston, Massachusetts, United States of America; 2 Department of Orthopaedic Surgery, Boston Children's Hospital, Harvard Medical School, Boston, Massachusetts, United States of America; 3 School of Medicine, Tufts University, Boston, Massachusetts, United States of America; 4 Department of Orthopaedic Surgery, University Hospital Basel and University of Basel, Basel, Switzerland; University of Rochester, United States of America

## Abstract

**Background:**

High resolution μCT, and combined μPET/CT have emerged as non-invasive techniques to enhance or even replace dual energy X-ray absorptiometry (DXA) as the current preferred approach for fragility fracture risk assessment. The aim of this study was to assess the ability of µPET/CT imaging to differentiate changes in rat bone tissue density and microstructure induced by metabolic bone diseases more accurately than current available methods.

**Methods:**

Thirty three rats were divided into three groups of control, ovariectomy and vitamin-D deficiency. At the conclusion of the study, animals were subjected to glucose (^18^FDG) and sodium fluoride (Na^18^F) PET/CT scanning. Then, specimens were subjected to µCT imaging and tensile mechanical testing.

**Results:**

Compared to control, those allocated to ovariectomy and vitamin D deficiency groups showed 4% and 22% (significant) increase in ^18^FDG uptake values, respectively. DXA-based bone mineral density was higher in the vitamin D deficiency group when compared to the other groups (cortical bone), yet μCT-based apparent and mineral density results were not different between groups. DXA-based bone mineral density was lower in the ovariectomy group when compared to the other groups (cancellous bone); yet μCT-based mineral density results were not different between groups, and the μCT-based apparent density results were lower in the ovariectomy group compared to the other groups.

**Conclusion:**

PET and micro-CT provide an accurate three-dimensional measurement of the changes in bone tissue mineral density, as well as microstructure for cortical and cancellous bone and metabolic activity. As osteomalacia is characterized by impaired bone mineralization, the use of densitometric analyses may lead to misinterpretation of the condition as osteoporosis. In contrast, µCT alone and in combination with the PET component certainly provides an accurate three-dimensional measurement of the changes in both bone tissue mineral density, as well as microstructure for cortical and cancellous bone and metabolic activity.

## Introduction

 Fragility fractures occurring at multiple skeletal sites affect approximately 1.5 million in the United States annually [1], where four out of ten white women aged 50 or older will suffer a fracture due to osteoporosis. Most commonly involved sites include the spine, the distal forearm and the hip [2,3]. The projected increase of the elderly population will most likely increase in number of fractures following falls in those at greater risk [4]. Fragility fractures in osteoporosis are caused by low bone mass and micro-architectural deterioration of bone tissue [5]. The World Health Organization identifies individuals at risk for these types of fractures based on their areal bone mineral density (aBMD) measured by dual energy X-ray absorptiometry (DXA) at the hip, lumbar spine or forearm, compared to normal reference values [6]. However, such aBMD-based fracture predictions have been shown to lack sensitivity and specificity [7-11].

 While osteoporosis is assumed to be the cause of most fragility fractures, 25-OH-vitamin D deficiency (a condition not distinguished by densitometric measurements), is observed in more than half of postmenopausal women receiving osteoporosis therapy [12]. Vitamin D deficiency results in osteomalacia in adults. A positive association has been found between 25-OH-vitamin D levels and total hip aBMD; and correspondingly, lower concentrations of this mineral have been reported in patients who sustained hip fractures, compared with controls [13,14]. Additionally, typical histological changes seen in osteomalacia were found in 25% of patients with femoral head fractures [15]. The increased local rate of bone turnover translates in decrements in aBMD and bone mineral content, changes that are more pronounced in cancellous bone [16]. Although it has been stated that aBMD values are not necessary for the diagnosis of osteomalacia, their serial measurement following initiation of treatment makes it a valuable tool. It has been shown that clinical and biochemical improvement ensues somewhat rapidly, while bone mineral deficit takes longer to correct.

 As they may coexist, we find relevant to establish significant biochemical and structural differences between osteoporosis and osteomalacia, as a means to provide better treatment options for each pathologic condition. The former is characterized by a loss of the integrity of the trabecular network and the cortical shell, resulting in overall reduction in bone strength [17]. In contrast, osteomalacia results due to a failure of the osteoid to calcify as it is laid down, leading to an increased osteoid surface and width, and decreased mineralizing surfaces [18]. While both conditions lead to alterations in bone structure and strength, osteoporosis occurs with relatively normal bone tissue density (bone mass/bone tissue volume, g.cm^-3^) and a significant decrease in bone volume fraction (bone tissue volume/total specimen volume, mm^3^/mm^3^). In contrast, osteomalacia presents with a significant decrease in bone tissue density and a less pronounced decrease in volume fraction [19]. 

 There are limitations associated with current diagnostic methods to differentiate between metabolic bone diseases that alter the mineral and structural states of cortical and cancellous bones through different mechanisms. In a previous study, we used several surrogate rat models to mimic altered skeletal states and showed the inability of DXA to differentiate changes occurring in bone microstructure or density [19]. In that study we highlighted the advantages of using volumetric methods, such as quantitative computed tomography (QCT), to provide an accurate assessment of changes in both bone mineral density and microstructure occurring in cortical and cancellous bone. However, although QCT provides more information than DXA, it still does not yield details regarding bone biology and its interaction with the tissue’s architecture and material properties. Getting such information gains more relevance when diagnosing and addressing the consequences of common metabolic conditions that compromise the normal skeletal homeostasis. 

 High-resolution liquid/solid state magnetic resonance imaging (MRI) and combined positron emission tomography (PET) and CT imaging can potentially assess bone biology and architecture. PET is a well-established, non-invasive imaging modality that evaluates metabolic activity through uptake of radioactive tracers in tissue. Historically, clinical PET and CT scanners have been hindered by low-resolution. The development of high-resolution scanners that combine both imaging modalities have recently allowed the critical study of bone. These novel techniques are providing a great deal of information related to the physiological and architectural responses of bone to aging and treatment. Furthermore, the expanding clinical usage of PET/CT has recently increased the interest of using ^18^F-fluoride PET/CT for bone imaging [20]. This ion has been widely recognized for its ability to deposit in bone, preferentially at the sites of high osteoblastic activity related to bone remodeling [21,22].

 More recently, three-dimensional high-resolution peripheral quantitative computed tomography (HR-pQCT) provides the ability to similarly measure components of bone quality such as BMD, micro-architectural morphology and bone mechanics. The increased spatial resolution underscores its clinical importance, allowing the *in vivo* assessment of bone quality, a well-known limitation of currently used techniques [23,24]. 

 The ovariectomized rat model has been widely used to study the effects of menopause on bone mass, trabecular microstructure and fracture risk [25-30]. Similarly, the chronic vitamin D deficient diet rat model has been used to assess the burden of osteomalacia on bone metabolism [29,31]. We used the established ovariectomy model in the present study as a surrogate for osteopenia, in conjunction with the vitamin D deficient diet model as a surrogate for osteomalacia. Both models aim to induce altered states of bone mineralization/microarchitecture to enable us to study the performance of combined PET/CT imaging to assess changes in bone tissue density and microstructure, as compared to current gold standard techniques of DXA, as well as high-resolution CT imaging, and mechanical testing. 

 To this end, *we hypothesize that µPET/CT imaging is capable of assessing changes in rat bone tissue density and microstructure induced by metabolic bone diseases more accurately than current available methods*. By providing a more detailed analysis, information obtained from this approach could ultimately lead to better bone quality assessment as well as accurate non-invasive diagnosis of specific metabolic bone diseases.

## Materials and Methods

### Animal Models

**Figure 1:**
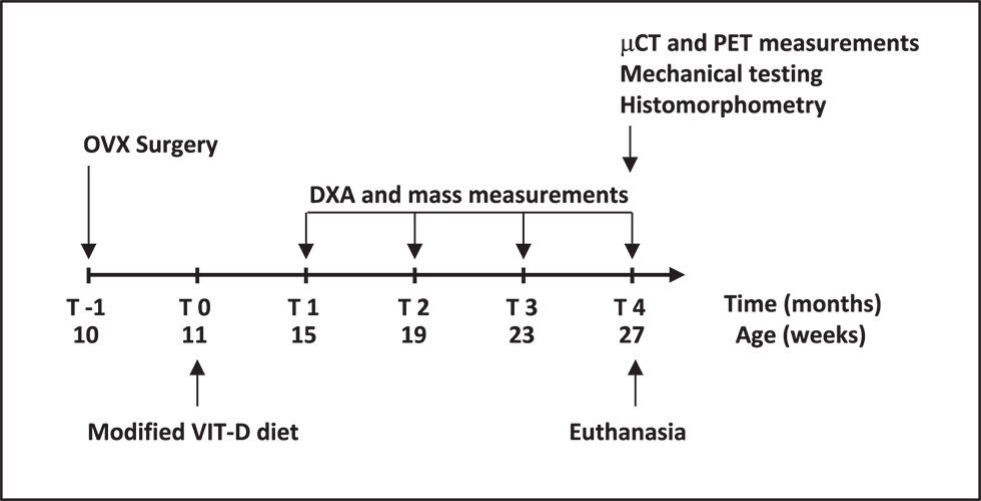
Study time-line highlighting surgical procedure and measurements.

 Thirty three female Sprague Dawley rats (11 weeks old, mass: 200-225 grams) were obtained from Charles River Laboratories (Charles River, Charlestown, MA, USA) and divided into three equally sized groups. The animals in the control group (CON) were not subjected to any surgical or dietary interventions. The animals allocated to the OVX group underwent ovariectomy to induce a state of low bone mass and micro-architectural deterioration. Finally, the animals assigned to the vitamin D deficient VIT-D group were placed on a modified diet to induce inadequate bone mineralization. The ovariectomy procedure was conducted at the animal supplier facility one week prior to the arrival of the animals to our laboratory. The vitamin D deficient diet consisted of 0.4% calcium and 0% vitamin D (modified Basal Diet 5755, TestDiet, Richmond, IN, USA). All animals underwent monthly measurement of bone mineral content and density via DXA. They were also weighted in the same interval to assess changes in body mass over the study period. At the conclusion of the study period (4 months following arrival at our laboratory), the animals were subjected to ^18^fluoro-2-deoxyglucose (FDG) and Na^18^F PET/CT scanning. Following the image acquisition protocol, all rats were euthanized by means of CO_2_ gas inhalation. Femurs were excised and cleaned from adherent soft tissue. One femur from each animal, selected at random, was subjected to micro-CT (µCT) imaging and later underwent destructive mechanical testing (Figure **1**). The study protocol was approved by Beth Israel Deaconess Medical Center’s Institutional Animal Care and Use Committee (IACUC).

### In-vivo Dual Energy X-ray Absorptiometry (DXA)

Areal bone mineral density (aBMD, g.cm^-2^) and bone mineral content (BMC, g) were measured from the femoral diaphysis (cortical bone) and distal metaphysis (cancellous bone) on a monthly basis over a four-month period. Measurements were conducted using a pre-clinical DXA system (Lunar PIXImus2, General Electric, Waukesha, WI, USA). Two landmark-based analysis boxes, one to cover the cortical bone area and the other to cover the distal femoral metaphysis, were used to assess aBMD and BMC throughout the study.

### In-vivo Micro Positron-emission Tomography/Computed Tomography (µPET/CT)

 µPET/CT scanning was performed on five animals per group using a mosaic high performance µPET (Philips, Cleveland, OH, USA) in conjunction with the µCT component of a nano-SPECT/CT system (Bioscan, Washington DC, USA). The µPET system achieves a 1.9 mm full-width half-maximum (FWHM) spatial resolution and an 11.9 cm axial field-of-view. Rapid reconstruction of the final three-dimensional data set was performed on the Imalytics Workspace (Philips, Cleveland, OH, USA) for high throughput and quantitative data analysis. Animals were scanned over a two-day period, with 18FDG PET and CT imaging (450 µm voxel size) conducted on day one, followed by Na18F PET imaging on day two. Bone density was assessed using water and air signals for calibration. One femur from each animal was contoured to assess the percentages of 18FDG and Na18F uptake in a specific anatomic location. 

### Ex-vivo Micro-computed Tomographic Imaging (µCT) and Image Analysis

Sequential transaxial images through the entire diaphyseal cortical and metaphyseal trabecular sections of the femurs were obtained using µCT at an isotropic voxel size of 30 µm, integration time of 250 ms, tube voltage and current of 55 KVp and 145 µA respectively, while applying a 1200 mg.cm^-3^ hydroxyapatite beam hardening correction (µCT 40, Scanco Medical AG, Brüttisellen, Switzerland).

Images were binarized to separate bone from background using an established adapting thresholding procedure [32]. A three-dimensional Gaussian filter (σ = 0.8) with a limited, finite filter support (support = 1) was used to suppress the noise in the volumes. In this technique, the optimal threshold is chosen automatically as a result of an iterative process, where successive iterations provide increasingly cleaner extractions of the object region. 

After thresholding, the following parameters were assessed for all images [33]. Cortical bone volume fraction (Ct.BV/TV); cortical thickness (Ct.Th), along with direct trabecular morphometric indices of bone volume fraction (BV/TV); bone surface density (BS/BV); structure model index (SMI); trabecular number (Tb.N); trabecular thickness (Tb.Th) and spacing (Tb.Sp), each with its intra-individual standard deviation (Tb.Th SD and Tb.Sp SD, respectively), calculated as the second moment of each’s histogram distribution; degree of anisotropy, defined as the longest divided by the shortest mean intercept length (DA); and connectivity density (Conn.D). Additionally, cortical and cancellous volumetric bone mineral density (σ_MIN_, g.cm^-3^) and apparent bone density (σ_APP_, g.cm^-3^) were measured using a hydroxyapatite phantom, supplied by the manufacturer, to convert the X-ray attenuation coefficient (µ) to mineral density. The variability of µCT assessment of three-dimensional microstructural and densitometric indices of excised rat bone samples is less than 0.5% at our laboratory. 

### Ex-vivo Tensile Mechanical Testing

**Figure 2:**
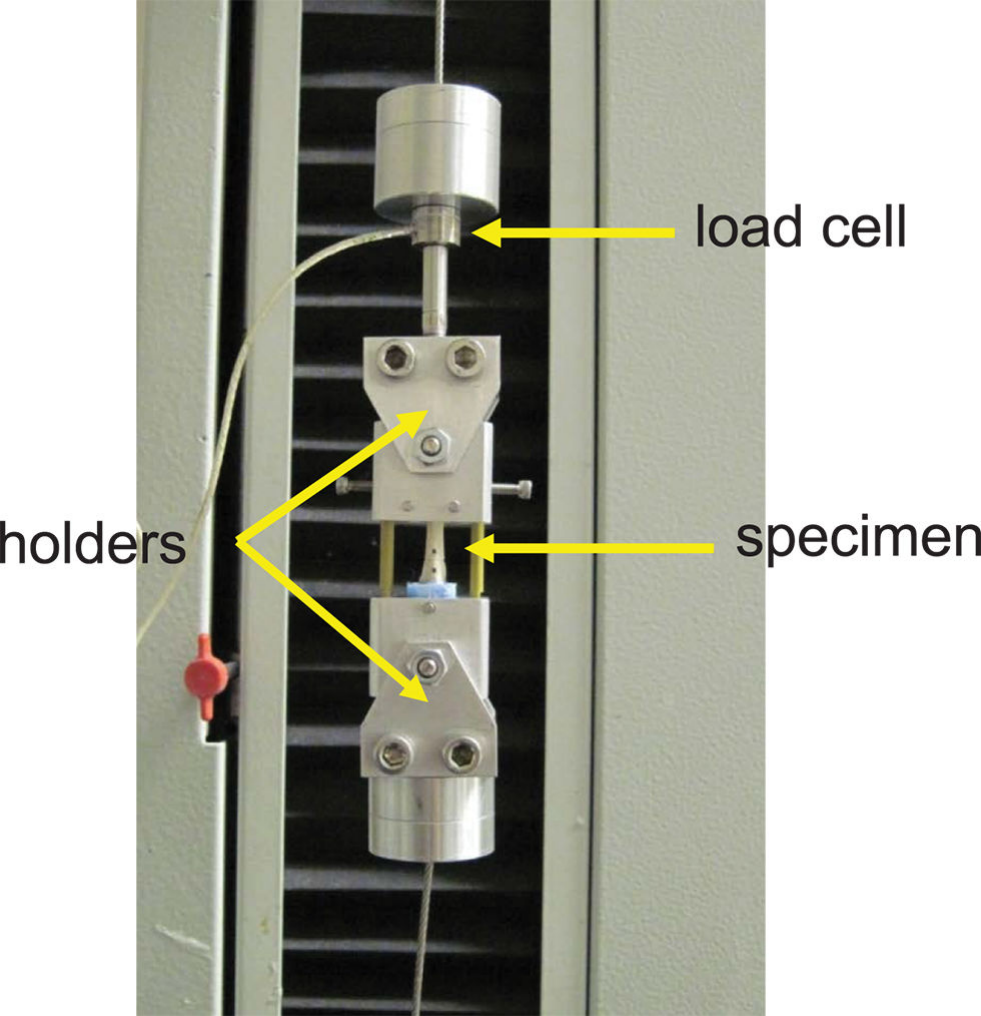
An illustration of the tensile testing mechanism used in the study.

 Following the µCT imaging, both ends of the femurs were embedded in polymethylmethacrylate to provide adequate gripping surfaces for mechanical testing. A stress riser notch was placed at the mid-diaphysis of each femur with markers placed at each side used as reference points for the camera to be able to assess mid-axis strain during testing. The prepped femur was mounted into a previously described tensile apparatus [34] and was loaded to failure at a strain rate of 0.005 s^-1^ (Synergy 200, MTS Systems, Eden Prairie, MN, USA) (Figure **2**). A digital camera, which was previously calibrated with a calibration glass, (PixeLINK CMOS microscopy camera model PL-B681C, Ottawa, ON, Canada) captured images at a rate of 20 frames/s throughout testing. A Matlab program (MATLAB v12.0, Mathworks, Natick, MA, USA) was used to determine the mid-axis strain by measuring the differences in distances between the centroids of the markers in each image (positioned above and below the stress riser notch). The cross-sectional area of bone for each slice was calculated from the µCT images by counting the number of bone voxels and multiplying by pixel size. In cortical bones, the cross-section with the minimum bony area was used to calculate the tensile strength. Additionally, extrinsic tensile properties of the cortical component of the femurs, such as tensile modulus (E, MPa), ultimate strain (ε_ULT_, mm/mm), ultimate strength (σ_ULT_, MPa), and energy to failure (Energy, J) were calculated for all specimens. 

### Statistical Analysis

 Normality of continuous data was assessed using the Kolmogorov-Smirnov test. Power analysis indicated that a sample size of 11 animals randomized to each of the three groups would provide 80% power to detect a 10% difference in FDG uptake (assuming a standard deviation of 6%, effect size = 1.67) using the F-test in ANOVA  with a two-tailed Bonferroni-corrected p < 0.05 (nQuery Advisor, Statistical Solutions, Saugus, MA). Different parameters including aBMD, BMC, body mass, cancellous and cortical bone microstructural indices, bone tissue density (σBONE), PET 18FDG, Na18F, PET HU, and extrinsic mechanical properties served as dependent variables and were compared across the three groups with the use of a one-way analysis of variance [35]. Also, a one-way ANOVA with post-hoc Bonferroni correction for multiple comparisons was conducted with aBMD, BMC, body mass and femur length over time as dependent variables. 

 Data analysis was performed on the SPSS statistical package (version 19.0, Chicago, IL, USA). All reported p-values are two-tailed with p<0.05 considered statistically significant. 

## Results

**Figure 3:**
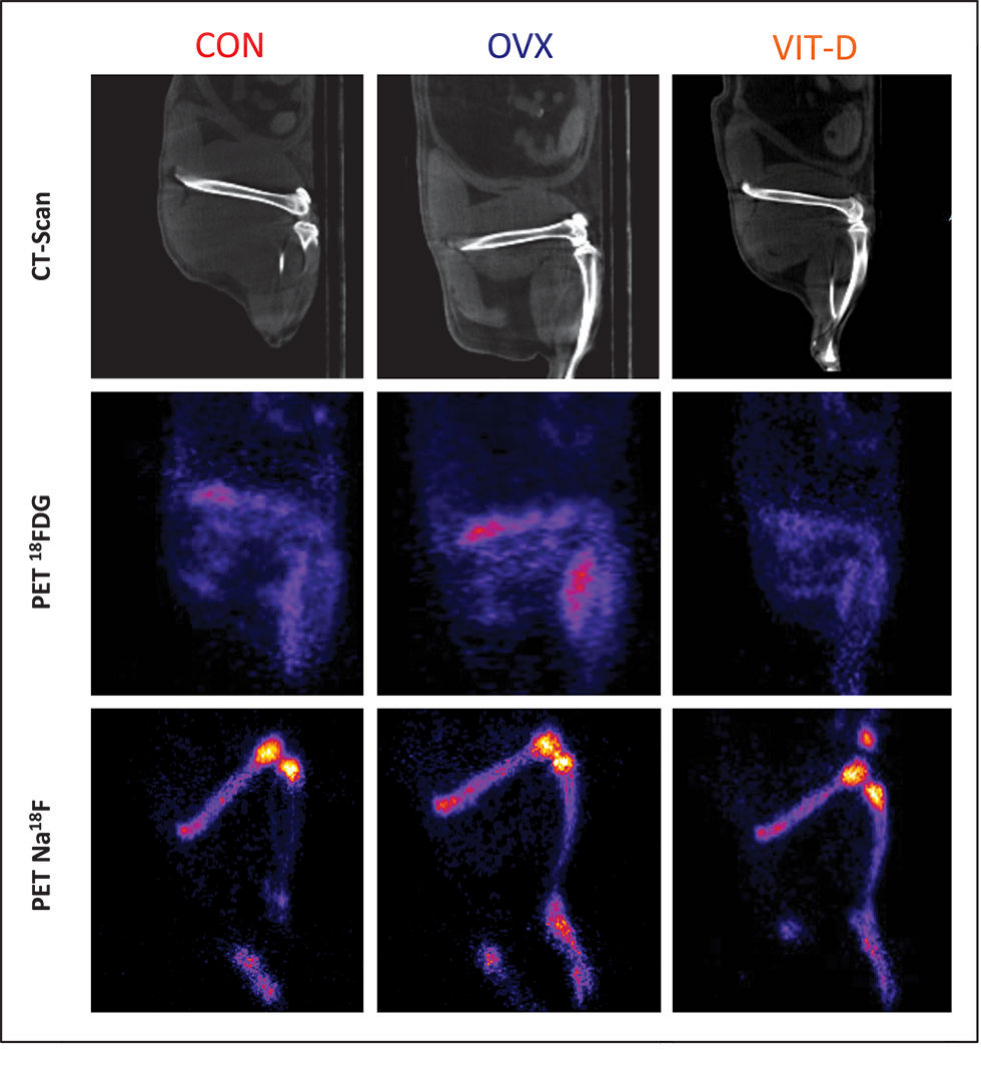
CT, PET ^18^FDG and PET Na^18^F images from representative animals from CON, OVX and VIT-D groups.

The ^18^FDG PET imaging intent was to quantify the metabolic activity of the bone, whereas the Na^18^F PET imaging aimed to quantify the osteoblastic activity within the bone. Compared to the control, those allocated to the OVX and VIT-D groups showed a relative increase of 4% (P = 0.823) and 28% (P = 0.015) in ^18^FDG uptake values, respectively (Table **1** and Figure **3**). The VIT-D group exhibited an increase 17% uptake when compared to the OVX group (P = 0.031). Additionally, the experimental groups showed lower, yet non-significant, Na^18^F uptake values when using the control group as a reference (4% (0.0775) and 8% (P = 0.403) for OVX and VIT-D, respectively). Bone density estimated in Hounsfield units from the PET/µCT images showed that the CON group had relatively higher values when compared to those in the OVX and VIT-D groups P=0.0001 for both cases). Also, the OVX group indicated higher bone density values than the VIT-D group (P = 0.0001). 

**Table 1:** PET/CT based 18FDG and Na18F uptake and bone density values for femurs.

	PET/CT-based Measurements		
Group	FDG Uptake [%]	NaF Uptake [%]	Bone Density [HU]
CON	68.1	100	1325
*Std Dev*	5.3	-	24.7
OVX	70.9	95.7	1138
*Std Dev*	6.6	6.8	17.5
VIT-D	87.7	92.0	1090
*Std Dev*	5.9	8.1	13.3
P [Group]	**0.013**	0.433	**0.0001**
P*: CON-OVX	0.823	0.775	**0.0001**
P*: CON-VIT-D	**0.015**	0.403	**0.0000**
P*:OVX-VIT-D	**0.031**	0.775	**0.023**

P*: P values from Bonferonni post-hoc analysis

^*^ CON NaF values were used as reference (%100) for other groups.

 There were no differences in the DXA-based cortical BMC values between the three study groups; however, disparities were seen in the aBMD values when comparing rats in the VIT-D groups with those in the CON and OVX groups (p=0.01) (Table **2**). Regarding mechanical testing-based measurements, cortical bone ultimate strength was significantly lower in the OVX and VIT-D groups (p<0.01) in comparison to that of the control. No differences were observed in energy to failure. However, specimens from the OVX group showed lower tensile modulus values when compared to the other two groups, only reaching statistical significance against the CON group (p<0.01). Although none of the µCT-based cortical bone parameters reached a statistically significant difference, this was not the case for cancellous bone. BMC and aBMD were significantly lower in the OVX group as compared to the other two groups (p<0.001) (Table **3**). The OVX group showed significantly lower values in comparison to the other two categories in parameters such as bone volume fraction, connectivity density, trabecular number, and apparent density (p<0.001). As for trabecular spacing, the OVX group showed significantly higher values (p<0.001). The structure model index was different across all three groups (p<0.05), while the degree of anisotropy was higher in the CON group only when comparing individually against the OVX and VIT-D groups (p<0.001 for both). 

**Table 2:** μCT based morphometric indices, mechanical results and DXA based densitometric values for cortical bone specimens.

	uCT-based Measurements					Mechanical Testing-based Measurements				DXA-based Measurements	
Group	BV/TV [mm^3^/mm^3^]	BS/TV [mm^3^/mm^3^]	Cort.Th [mm]	App. Density [g/cm^3^]	Bone Density [g/cm^3^]	Ult. Strain [mm/mm]	Ult. Strength [MPa]	E [MPa]	Energy to Failure [kJ]	BMC [g]	aBMD [g/cm^2^]
CON	0.66	3.27	0.61	741.96	1110.73	0.015	24.35	1583.14	0.12	0.151	0.191
*Std Dev*	*0.02*	*0.16*	*0.03*	*34.34*	*13.26*	*0.01*	*6.08*	*439.44*	*0.08*	0.011	0.018
OVX	0.62	3.09	0.65	708.89	1098.70	0.02	15.82	869.38	0.12	0.158	0.191
*Std Dev*	*0.02*	*0.18*	*0.04*	*24.38*	*8.33*	*0.01*	*5.85*	*300.14*	*0.08*	0.013	0.019
VIT-D	0.65	3.27	0.61	729.55	1103.12	0.01	14.40	1268.63	0.07	0.151	0.202
*Std Dev*	*0.04*	*0.18*	*0.04*	*48.64*	*17.62*	*0.00*	*4.39*	*322.10*	*0.03*	0.006	0.014
P [Time]										**0.001**	**0.001**
P [Group]	**0.060**	**0.040**	**0.040**	0.150	0.150	**0.010**	**0.001**	**0.001**	0.140	0.992	**0.003**
P*: CON-OVX	**0.070**	0.080	0.080	0.170	0.170	0.110	**0.005**	**0.001**	0.990	0.990	0.990
P*: CON-VIT-D	0.990	0.990	0.990	0.990	0.660	0.920	**0.001**	0.180	0.280	0.990	**0.010**
P*:OVX-VIT-D	0.190	0.090	0.100	0.670	0.990	**0.010**	0.990	**0.058**	0.230	0.990	**0.007**

P*: P values from Bonferonni post-hoc analysis

**Table 3:** μCT based morphometric indices and DXA based densitometric values for cancellous bone specimens.

	uCT-based Measurements													DXA-based Measurements	
Group	BV/TV [mm^3^/mm^3^]	BS/BV [mm^2^/mm^3^]	Conn.D. [1/mm^3^]	SMI	Tb.N [1/mm]	Tb.Th [mm]	Tb.Sp [mm]	Tb.1/N SD	Tb.Th SD	Tb.Sp SD	App. Density [g/cm^3^]	Bone Density [g/cm^3^]	DA	BMC† [g]	aBMD† [g/cm^2^]
CON	0.235	34.35	110.26	1.78	4.27	0.075	0.24	0.114	0.02	0.12	316.01	927.83	1.69	0.083	0.212
*Std Dev*	*0.039*	*6.27*	*39.04*	*0.40*	*0.97*	*0.01*	*0.07*	*0.06*	*0.005*	*0.06*	*34.97*	*34.46*	*0.08*	0.009	0.021
OVX	0.082	34.69	24.79	2.43	1.28	0.07	0.84	0.61	0.02	0.61	144.04	927.71	1.47	0.075	0.199
*Std Dev*	*0.01*	*2.15*	*5.91*	*0.17*	*0.33*	*0.04*	*0.20*	*0.17*	*0.00*	*0.17*	*19.28*	*15.11*	*0.10*	0.006	0.016
VIT-D	0.25	29.31	92.53	1.35	3.79	0.08	0.27	0.14	0.02	0.15	338.07	932.68	1.41	0.081	0.216
*Std Dev*	*0.06*	*3.09*	*21.39*	*0.41*	*0.83*	*0.00*	*0.08*	*0.07*	*0.00*	*0.07*	*61.33*	*17.60*	*0.05*	0.007	0.019
P [Time]														**0.001**	**0.001**
P (ANOVA)	**0.001**	**0.020**	**0.001**	**0.001**	**0.001**	0.170	**0.001**	**0.001**	0.720	**0.001**	**0.001**	0.890	**0.001**	**0.001**	**0.001**
P*: CON-OVX	**0.001**	0.990	**0.001**	**0.002**	**0.001**	0.990	**0.001**	**0.001**	0.990	**0.001**	**0.001**	0.990	**0.001**	**0.001**	**0.001**
P*: CON-VIT-D	0.990	0.059	0.502	**0.040**	0.590	0.220	0.990	0.990	0.990	0.990	0.810	0.990	**0.001**	0.440	0.990
P*:OVX-VIT-D	**0.001**	0.056	**0.001**	**0.001**	**0.001**	0.540	**0.001**	**0.001**	0.990	**0.001**	**0.001**	0.990	0.990	**0.001**	**0.001**
									0.99						

P*: P values from Bonferonni post-hoc analysis

^†^ BMD values reported from the last time point

**Figure 4:**
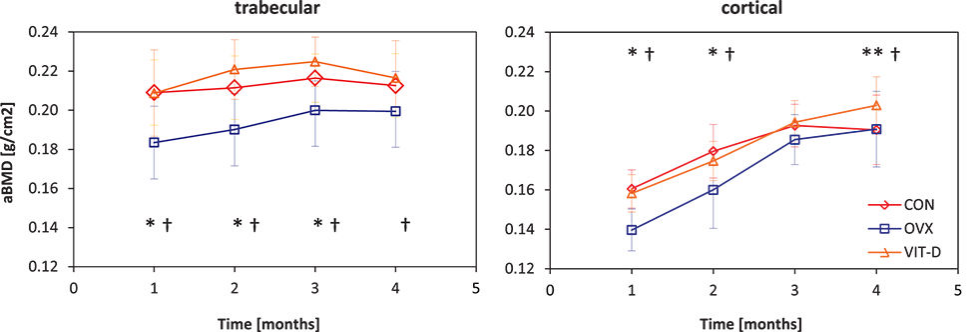
DXA-based aBMD changes over the 4 months study period in CON, OVX and VIT-D groups in both trabecular and cortical bone tissues (* indicates significance between CON and OVX groups, ** denotes significance between CON and VIT-D groups, † indicates significance between the OVX and VIT-D groups).

 Over the course of the study, cortical aBMD measurements of the two experimental groups showed an upward trend; while the control group exhibited a fairly linear trend between time-points 3 and 4 (Figure **4**). Cancellous aBMD trends demonstrated a rather linear pattern with overall subtle decreasing changes at the end of the study period in the CON and VIT-D groups.

**Figure 5:**
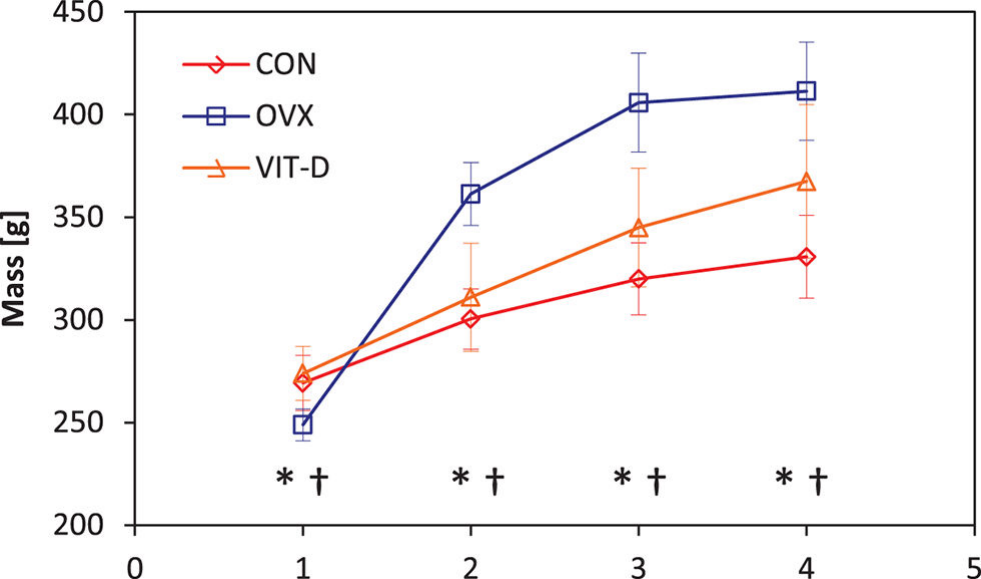
Body mass changes over the 4 months study period in CON, OVX and VIT-D groups (* indicates significance between CON and OVX groups, ** denotes significance between CON and VIT-D groups, † indicates significance between the OVX and VIT-D groups).

 All animals gained weight during the study period. Animals in the OVX group exhibited the greatest gain, especially during the first two time points, where a dramatic slope was seen. Although no greater than the OVX group, those in the VIT-D group gained more weight than the control group as well (Figure **5**).

## Discussion

We aimed to prove the ability of combined µPET/CT imaging to effectively detect bone tissue changes in the presence of metabolic diseases that promote an altered skeletal state. Although bone turnover markers measured in serum and urine can aid in the diagnosis of metabolic bone diseases, bone histomorphometry is still considered the gold standard method. However, given the invasive nature of the procedure, novel and reliable non-invasive methods have been long sought for diagnosing and evaluating treatment of patients affected with these bone conditions [22]. The role of bone mass loss in the pathophysiology of fragility fractures has taken most of the attention; however, we now know that bone quality plays an even more crucial role. The concept of quality comprises multiple aspects that include material composition and property, geometry, cellularity, bone turnover, as well as mineralization, microarchitecture and microdamage [36-38]. As portrayed by previous authors [38], densitometric analysis fails to gather many of the aforementioned parameters, thus limiting its assessment to bone’s quantity, rather than its quality. 

### µPET/CT Imaging Analysis

Results of the µPET/CT imaging aimed at quantifying the metabolic activity of the bone illustrated that the experimental groups displayed higher ^18^FDG uptake values. Several factors seem to play a role in the accumulation of tracers in areas where microdamage is present, such as increased exposure of the mineral surface favored by the presence of cracks, increased blood flow and vascular permeability, and active osteoblastic differentiation and proliferation due to microdamage repair [39,40]. Li et al [36] were able to show fairly good correlation values between bone microdamage as shown by µPET/CT and histomorphometry analyses in ovariectomized rat models. However, in the latter case, microdamage was induced by fatigue loading following estrogen depletion. Kato et al [41] proposed the use of FDG-PET analysis to distinguish benign from malignant metastatic fractures. Curiously, they concluded that acute benign fractures do not show significant tracer uptake values. Similarly, Schmitz et al [42] found that acute vertebral fractures originated from osteoporosis or preclinical osteoporosis, tended to have no pathologically increased FDG uptake. On the other hand, Na^18^F uptake values showed a different and downward trend in the experimental groups. In this case, the OVX and VIT-D groups displayed slightly lower uptake values than those seen in the control group. Similar results were seen in a study conducted by Wang et al [43], in which a significant decrease in ^18^F uptake was seen in osteoporosis rat models induced by excess of dexamethasone phosphate sodium injections. The same decreased uptake pattern was evidenced in a human study by Uchida et al [44]. To our knowledge, analysis of tracer uptake in vitamin D deficient rats had not been conducted prior to our study, showing a similar decreased uptake behavior. The tracer kinetics of this ion is higher at sites of high bone turnover and remodeling, which leads to interpretation of our results as less bone formation in the experimental groups [45]. The increased sensitivity to molecular details gives µPET/CT imaging several advantages over other methods, mainly the ability to estimate the activity of osteoblasts and osteoclasts within the tissue, as well as to distinguish the effects of mechanical loading and bring into evidence newly-developed microdamage [40]. However, its expensive cost - more so when combining both PET and CT - and the relatively short half-life of commonly used radioisotopes accounts for some of the disadvantages to be taken into consideration. Our findings call for further research addressing the differences in ^18^F uptake in ovariectomized models, as well as potential disparities in such measurements when inducing micro-damage or not. 

### DXA Imaging and Mechanical Testing

After analyzing different parameters using pathological models that alter the bone’s structure in many levels, we were able to identify the effect these changes have in cortical and cancellous bone. Cortical bone DXA analysis only showed differences regarding higher aBMD values in the VIT-D group as compared with the OVX and CON groups (table **2**). Although we expected the VIT-D rats to show lower bone density values as compared to the control group, our findings did not portray such results [46]. Nonetheless, these findings, as well as those obtained from µCT analysis, suggest that there is minimal compromise of cortical bone when affected by disease states addressed in our study. Although not statistically significant, specimens from the OVX group showed slightly higher cortical thickness values when compared to the other two groups. This could be explained by a small compensatory effect occurring in cortical bone as a result of the pathological changes taking place in the trabecular tissue [19,47]. As expected, mechanical testing-based measurements showed lower ultimate strength values in the experimental groups as compared to the control. Yet, no differences in energy to failure were observed across groups, and only the OVX specimens showed significantly lower tensile moduli. These results from cortical bone analysis support findings from one of our previous works [19], which showed that DXA and μCT imaging were unable to show important changes in the material, structural, and mechanical properties of femoral cortical bone in the ovariectomized model, at least 7 weeks following ovariectomy. Similarly, the inconsistency of densitometric measurements and mechanical testing parameters attest to the interaction of multiple aspects that govern the structural and mechanical properties of bone and its concomitant changes in the tissue’s biomechanics when affected by different pathological processes. Skeletal diseases cause fragile bones affecting its structure through different mechanisms [37]. While osteomalacia results in weak and ductile bones, osteoporosis reduces bone formation, both resulting in reduced ultimate strength. Following this analysis, it is evident how the traditional non-invasive densitometric and CT imaging techniques failed equally to detect the strength differences seen in cortical bones’ mechanical testing across the three groups. Nonetheless, this was not the case when assessing cancellous bone. 

DXA-based measurements only picked up significantly lower BMC and aBMD values in the OVX group when compared to the VIT-D and CON groups. Our measurements failed to detect differences when comparing CON with the VIT-D group. However, differences between the morphometric indices of the three groups were identified by µCT imaging and analysis. Bone volume fraction, connectivity density, trabecular number and apparent density values were lower in the OVX group when compared to the other two groups, yet there were no differences between the latter. As for trabecular spacing, rats from the ovariectomized group showed significantly higher values as compared to the VIT-D and control groups. The obtained values coincide with those portrayed by Dempster et al [48], who explained changes in cancellous bone due to estrogen deficiency by the osteoclast perforation of trabecular plates, resulting in decreased connectivity. In that case, which was also seen in this study, no generalized trabecular thinning ensued, thus no differences in trabecular thickness values were seen among either of the groups. As for the degree of anisotropy, those allocated to the CON group showed greater values than those from the experimental groups. Thus, specimens from the OVX and VIT-D groups moved towards a more isotropic yet undesirable state, detrimental to the multi-axial loading of bone during daily activities. SMI values differed significantly across the three groups, with OVX showing the highest measurement, followed by CON and then the VIT-D group. This measurement allows us to quantify the characteristic form of a three-dimensional structure in terms of the amount of plates and rods composing the structure [49]. Disregarding the physical dimensions, ideal plate and rod structures will show values of 0 and 3, respectively. Consequently, for a structure with both plates and rods in equal proportions, the value will fall in the middle of these values. Cancellous bone from the OVX and VIT-D groups moved in opposite directions, the former showing a predominantly rod-like pattern, whereas the latter more of a plate-like one. The increased values of both trabecular spacing and SMI in the ovariectomized rats suggests the occurrence of perforation of the trabecular plates, as shown in prior transmenopausal human studies by Akhter et al [50].

There is a consensus that ovariectomy causes rapid changes in cancellous bone, while changes in cortical bone are much slower [48,51]. Also, estrogen deficiency does not usually cause mechanical weakening of cortical bone [52]. These findings support the results seen in our study, confirming that ovariectomy induces high bone turnover that affects cancellous bone in an earlier fashion than cortical bone. Interestingly, although the OVX group showed lower aBMD values when compared to the controls, the trend shows somewhat an increase over time. We think this may be due to the acute changes seen following the abrupt estrogen depletion that induces a rapid and sudden change in aBMD that later stabilizes due to homeostatic adaptive changes made by the tissue in order to find equilibrium. Although we were not able to detect bone mineral density loss in the VIT-D group, a higher bone turnover and decreased bone mineralization is expected in the presence of vitamin D deficiency [53]. 

## Conclusion

We comprehensively assessed the differences across bones affected with different altered skeletal states induced by ovariectomy and vitamin D deficient diets using traditional as well as novel diagnostic modalities. Our findings further justify the inaccuracy of densitometric-based parameters in differentiating metabolic bone diseases. However, although beyond the scope of our study, its usefulness in the diagnosis of metabolic bone diseases should not be disregarded. DXA is still the most widely used method that has among its advantages the ability to obtain measurements at or close to a specific site of interest (commonly lumbar spine and proximal femur) [54,55]. Also, it has been recognized as being relatively less complex and inexpensive when compared to the other methods discussed in the present work [17]. However, DXA measures the average bone mineral content in a two-dimensional projected area with low spatial resolution, thus it cannot differentiate whether the changes occur in the bone microstructure or bone tissue density. Another known drawback of DXA is the possibility of overestimating aBMD values in posteroanterior measurements of the lumbar spine, due to the presence of typical degenerative changes seen in the elderly. Determination of volumetric bone mineral density in any part of the skeletal structure accounts for one of the main contrast points between QCT and DXA. Also, QCT allows for the possibility of assessing cortical and cancellous bone separately [56]. Although QCT has not proven to be superior to anteroposterior spinal DXA measurements, it eliminates artifacts from soft tissue and posterior elements previously mentioned that may overestimate some of the obtained values [57]. On the downside, the precision of QCT-derived BMD measurements depends in part of the repositioning of the patient, as well as in the size and shape of the region of interest. Also, the accuracy of such technique is limited by beam-hardening and partial volume averaging [58].

 As osteomalacia is characterized by impaired bone mineralization, the use of densitometric analyses may lead to misinterpretation of the condition as osteoporosis [59]. In contrast, CT imaging in combination with the PET component certainly provides an accurate three-dimensional measurement of the changes in both bone tissue mineral density, microstructure and metabolic activity for cortical and cancellous bone. 
